# Oncologic safety of autologous fat grafting in head and neck cancer patients: A scoping review

**DOI:** 10.4317/medoral.27795

**Published:** 2025-11-22

**Authors:** Martn Andura Correas, Guillermo Chacn Ferrer, Javier Gonzlez Martn-Moro, Mara Jos Morn Soto, Jos Luis Cebrin Carretero

**Affiliations:** 1 Oral and Maxillofacial Surgery Department, Hospital Universitario La Paz. Madrid, Spain.

## Abstract

**Background:**

Autologous fat grafting (AFG) has become increasingly used in reconstructive surgery due to its accessibility, safety, and regenerative potential. In head and neck cancer (HNC) patients, however, concerns remain regarding its oncologic safety, particularly due to the presence of adipose-derived stem cells (ASCs), which may theoretically influence tumor recurrence. This scoping review aims to synthesize current evidence regarding the safety of AFG in this unique patient population.

**Material and Methods:**

The review was conducted following the PRISMA-ScR guidelines. PubMed and Scopus databases were searched up to December 2024 using the terms "autologous fat grafting," "lipofilling," "head and neck cancer," and "oncologic safety." Eligible studies included clinical or experimental works reporting on AFG in HNC patients with oncologic outcome assessment. Exclusion criteria included purely aesthetic procedures and studies without relevance to oncologic safety. Data were extracted on study design, patient population, fat grafting technique, follow-up, and reported oncologic outcomes.

**Results:**

Five key studies fulfilled the inclusion criteria. Across these works, no evidence of increased recurrence or metastasis following AFG was reported. Favorable functional and aesthetic outcomes were consistently observed, although methodological variability and short follow-up durations limited the robustness of conclusions.

**Conclusions:**

Current evidence suggests that AFG is oncologically safe and beneficial for reconstructive purposes in head and neck cancer patients, improving contour and tissue quality. Nevertheless, the lack of large, prospective, and long-term studies precludes definitive recommendations. Standardized protocols and extended oncologic follow-up are essential to confirm safety and guide future clinical practice.

## Introduction

Head and neck cancer (HNC) comprises a diverse group of malignancies that arise from the mucosal linings of the oral cavity, pharynx, larynx, nasal cavity, and paranasal sinuses, as well as from salivary glands and skin of the head and neck region (1). Globally, head and neck squamous cell carcinoma (HNSCC) accounts for more than 90% of these cases, with an annual incidence exceeding 550,000 cases and approximately 300,000 related deaths per year. The aggressive nature of these tumors, along with their tendency to invade surrounding functional structures, necessitates multimodal treatment, typically involving surgical resection, radiation therapy, and chemotherapy (2).

While oncologic outcomes have improved with modern protocols, treatment-induced morbidity remains substantial. Surgical excision often results in contour defects, asymmetries, and functional deficits such as trismus, dysphagia, or speech impairment. Moreover, radiation therapy, a cornerstone of locoregional control, induces soft tissue fibrosis, lymphedema, and microvascular compromise, further exacerbating aesthetic and functional deformities. Reconstruction and rehabilitation following oncologic treatment have therefore become central to restoring patient quality of life (3).

Among the various reconstructive techniques available, autologous fat grafting (AFG)-also referred to as lipofilling-has gained significant popularity due to its accessibility, biocompatibility, and dual role in volume restoration and tissue regeneration. First introduced by Neuber in 1893 and refined by Coleman in the 1990s, AFG involves the harvest, processing, and reinjection of a patient's own adipose tissue into areas of soft tissue deficiency. It is especially appealing in the head and neck due to the complex anatomy and high cosmetic visibility of the region (4).

In aesthetic medicine, AFG is widely used and well-accepted. However, its application in oncologic reconstruction, particularly in the head and neck, remains controversial. The central concern revolves around the biological activity of adipose-derived stem cells (ADSCs), which are present in the stromal vascular fraction (SVF) of lipoaspirate. These multipotent cells exhibit regenerative effects by secreting growth factors and cytokines that promote angiogenesis, modulate immune response, and influence the extracellular matrix. Although these properties are beneficial in healing irradiated tissues and improving scar quality, they raise theoretical concerns regarding their interaction with residual or dormant cancer cells.

In vitro studies have shown that ADSCs can promote tumor cell proliferation, migration, and angiogenesis under specific experimental conditions. For instance, murine models and cell co-culture systems have demonstrated that ADSCs may enhance the aggressiveness of cancer cell lines, including breast and head and neck squamous carcinoma cells. This has led to a cautious stance among some clinicians regarding the oncologic safety of AFG in patients with a prior history of malignancy. The fear is that introducing a bioactive graft into a previously ablated oncologic field could theoretically stimulate dormant tumor cells and increase the risk of recurrence or metastasis.

Autologous fat grafting (AFG) has become an increasingly popular technique in reconstructive and symmetry (1 - 5). It involves the harvesting, processing, and reinjection of a patient's own fat tissue, providing a natural and biocompatible solution for soft tissue defects. Initially developed for aesthetic purposes, AFG has found extensive applications in the reconstructive setting, especially in patients who have undergone oncologic surgery in the head and neck region. This region is one of the most complex anatomical areas of the human body, where surgical resection of tumors often leads to significant aesthetic and functional deformities.

The use of AFG in head and neck cancer patients is particularly beneficial because of the regenerative properties of adipose tissue (1). Adipose tissue is not only a filler material but also a source of adipose-derived stem cells (ASCs), which have been shown to promote angiogenesis, enhance tissue healing, and reduce fibrosis (7). These regenerative properties have been leveraged to improve the outcomes of reconstructive surgery, leading to better aesthetic results and improved quality of life for patients. However, this same regenerative potential of ASCs has raised concerns regarding the oncologic safety of AFG, particularly in the context of cancer patients. Theoretical risks have been proposed, suggesting that ASCs may promote tumor growth either through direct stimulation of residual cancer cells or through paracrine signaling, which can create a more favorable environment for tumor proliferation.

Studies in the field of breast reconstruction have extensively explored the safety of AFG, generally finding no significant increase in cancer recurrence rates among patients undergoing AFG following mastectomy (7). However, the head and neck region presents unique challenges that distinguish it from other areas of the body. Head and neck cancers (HNCs) are a diverse group of malignancies arising from the mucosal surfaces of the upper aerodigestive tract, salivary glands, and thyroid. The anatomical complexity of this region, combined with the proximity of critical structures such as the facial nerve, blood vessels, and vital organs, makes the management of these cancers particularly challenging.

The theoretical risks associated with AFG in oncologic patients have been a subject of ongoing debate. Boschetti et al. (2023) conducted a multidisciplinary retrospective study focusing on the use of AFG following parotidectomy in head and neck cancer patients. The study reported favorable aesthetic outcomes with no significant increase in oncologic risks among patients who received AFG. This finding supports the notion that AFG can be safely applied in selected patients, provided that appropriate patient selection criteria and careful monitoring are employed (2).

Fiedler et al. (2021) explored this issue in a multinational study involving surgeons across four European countries. The study revealed that while many surgeons acknowledge the theoretical risks associated with AFG due to the presence of ASCs, most reported that they had not observed any cases of tumor recurrence associated with fat grafting in their practice. This discrepancy between theoretical risks and clinical observations highlights the need for a clearer understanding of the oncologic safety of AFG in head and neck cancer patients (1).

Similarly, Drochioi et al. (2019) emphasized the importance of careful patient selection and post-procedural monitoring when using AFG for craniofacial reconstruction in oncologic patients. The study highlighted the need for a standardized approach to patient selection, including the exclusion of patients with high-risk tumors or uncontrolled primary disease. Moreover, it underscored the necessity of regular imaging follow-up to detect any potential recurrence at an early stage (3).Karmali et al. published a retrospective series in which 116 patients were evaluated who underwent 190 autologous fat grafting procedures following oncologic head and neck reconstruction. The mean follow-up was 35.8 months. Six oncologic recurrences were reported during the observation period; however, none occurred at the grafted sites. The study concluded that AFG provided consistent esthetic improvement with low morbidity and did not compromise oncologic surveillance (4).

Griffin et al. (2019), in a retrospective cohort of 38 patients, showed lipotransfer was performed to address radiation-induced fibrosis and post-oncologic volume defects. With a mean follow-up of 32 months, 97% of patients demonstrated functional and esthetic improvement. Two oncologic recurrences were noted, both distant from the grafted areas. The authors highlighted significant gains in quality of life, supporting the safety and effectiveness of AFG in this context.

Despite the generally positive findings in the literature, the lack of standardized guidelines for the use of AFG in head and neck cancer patients remains a critical challenge. Different studies employ varying techniques for fat harvesting, processing, and injection, making it difficult to draw definitive conclusions regarding the oncologic safety of AFG. Furthermore, most studies are limited by small sample sizes and short follow-up periods, which may not be sufficient to fully assess the long-term safety of AFG in this patient population.

This scoping review aims to systematically explore the current evidence on the oncologic safety of AFG in head and neck cancer patients. By synthesizing findings from multiple studies, including the works of Boschetti et al. (2023), Fiedler et al. (2021), Drochioi et al. (2019), Karmali et al. (2018) and Griffin et al. (2019), this review seeks to provide clinicians with a clearer understanding of the benefits and risks of AFG in this unique patient population (1 - 5). In doing so, it aims to support the development of standardized guidelines and inform clinical practice.

PCC Framework for Research Question:

- Population (P): Patients with head and neck cancer

- Concept (C): Autologous fat grafting (AFG)

- Context (C): Oncologic safety

The research question guiding this scoping review was: In patients with head and neck cancer (Population), what is the evidence regarding the use of autologous fat grafting (Concept) in relation to oncologic safety (Context)?

## Material and Methods

Study Design

No prior protocol for this review was registered in databases such as PROSPERO or OSF. However, the study was designed and conducted in accordance with the PRISMA-ScR (Preferred Reporting Items for Systematic Reviews and Meta-Analyses extension for Scoping Reviews) guidelines to ensure methodological transparency and systematic approach to the literature search, selection, and synthesis.

Search Strategy

Five primary articles were selected for this review:

- Fiedler et al. (2021) examined the multinational trends and safety concerns of AFG in head and neck patients (1).

- Boschetti et al. (2023) explored the safety of AFG following parotidectomy in head and neck cancer patients (2).

- Drochioi et al. (2019) provided an overview of AFG in craniofacial reconstruction for oncologic patients (3).

- Karmali et al. examined a retrospective series of patients post-head and neck oncologic reconstruction (4).

- Griffin et al. analized a cohort of patients undergoing lipotransfer for radiation-induced fibrosis (5).

Eligibility Criteria

- Inclusion: Studies discussing AFG in head and neck cancer patients with a focus on oncologic safety.

- Exclusion: Studies not focused on cancer patients, or those where AFG was used for purely aesthetic purposes without oncologic consideration.

Data Extraction

Data from the included studies were extracted systematically, focusing on patient population, AFG techniques, follow-up duration, reported complications, oncologic outcomes, and recurrence rates.

Data Analysis

A narrative synthesis was employed, highlighting the findings of the included studies. The results are presented in a structured manner, following the key themes of oncologic safety, recurrence, and complications.

## Results

A total of five peer-reviewed studies met the eligibility criteria and were included in this scoping review. These sources comprised two retrospective cohort study (2, 5), a multinational clinician survey (1), a narrative review of fat grafting in oncologic craniofacial reconstruction (3) and a retrospective series of patients post-head and neck oncologic reconstruction. The characteristics and key findings of these studies are summarized below and stratified in the Table 1.

Table 1

Study Characteristics

Fiedler et al. (2021) performed a cross-sectional survey of 45 head and neck reconstructive surgeons from Germany, Austria, Switzerland, and the UK. The majority (62.2%) of respondents indicated that they use AFG in postcancer reconstruction, particularly to address radiation-induced fibrosis and facial contour defects. Interestingly, 47.6% of the surveyed surgeons did not routinely discuss the theoretical oncologic risk associated with fat grafting with their patients. Despite this, none reported cases of tumor recurrence attributable to AFG in their clinical practice. The survey highlighted variability in harvesting and processing techniques, with most clinicians relying on syringe aspiration and centrifugation methods. No consensus was found regarding best practices for oncologic patients (3).

Boschetti et al. (2023) conducted a multidisciplinary retrospective analysis of 30 patients who underwent parotidectomy for benign or malignant tumors. All patients received immediate reconstruction using autologous dermis-fat grafts harvested from the suprapubic region. Of the 30 patients, 8 had confirmed malignant neoplasms. The study reported no local recurrences or oncologic complications during the 12-month follow-up. Imaging via MRI confirmed stable reconstruction and allowed effective monitoring of the tumor bed. Cosmetic outcomes were deemed satisfactory in 26 of the 30 patients, with minor complications limited to hematoma formation and transient facial nerve weakness (2).

Drochioi et al. (2019) provided a comprehensive review of fat grafting in craniofacial oncologic patients, focusing on the regenerative potential of adipose tissue and associated risks. The authors discussed the unique properties of adipose-derived stem cells (ASCs), including their immunomodulatory and angiogenic functions. While acknowledging the theoretical risk of tumor stimulation based on in vitro studies, they emphasized that current clinical evidence does not support an increased risk of cancer recurrence. The review detailed surgical techniques, graft processing methods, and postoperative outcomes in oncologic reconstruction, concluding that AFG offers a viable and safe reconstructive strategy (3).

Karmali et al. in their retrospective series evaluated 116 patients who underwent 190 autologous fat grafting procedures following oncologic head and neck reconstruction. The mean follow-up was 35.8 months. Six oncologic recurrences were reported during the observation period; however, none occurred at the grafted sites. The study concluded that AFG provided consistent esthetic improvement with low morbidity and did not compromise oncologic surveillance (4).

Griffin et al. made a retrospective cohort of 38 patients, lipotransfer was performed to address radiation-induced fibrosis and post-oncologic volume defects. With a mean follow-up of 32 months, 97% of patients demonstrated functional and esthetic improvement. Two oncologic recurrences were noted, both distant from the grafted areas. The authors highlighted significant gains in quality of life, supporting the safety and effectiveness of AFG in this context (5).

Synthesis of Oncologic Outcomes

Across all five studies, no clinical evidence was found to suggest that AFG contributes to oncologic recurrence or metastasis in head and neck cancer patients. While the survey and narrative review noted the biological plausibility of tumor stimulation by ASCs, this risk remained speculative and unsupported by clinical observations. Only the retrospective cohort study offered direct outcome data, showing no oncologic events during one year of postoperative surveillance.

The limited number of high-quality studies, along with variability in protocols and short follow-up durations, underscores the need for more robust clinical data. Nonetheless, the convergence of findings across diverse study designs supports the preliminary conclusion that AFG does not increase the risk of cancer recurrence in this patient population.

## Discussion

The use of autologous fat grafting (AFG) in head and neck oncologic reconstruction represents an evolving intersection of reconstructive and regenerative medicine. This scoping review aimed to assess the oncologic safety of AFG in this context by synthesizing available clinical and experimental literature. Despite long-standing concerns regarding the potential tumor-promoting properties of adipose-derived stem cells (ADSCs), our review found no clinical evidence linking AFG to increased cancer recurrence. However, this absence of evidence should not be equated with conclusive proof of safety, particularly given the limited number and methodological scope of available studies.

Interpretation of Clinical Evidence

The most direct evidence regarding oncologic safety comes from the retrospective study by Boschetti et al., which followed 30 patients after parotidectomy with immediate dermis-fat grafting (2). Importantly, this study included patients with both benign and malignant lesions, thereby offering a real-world snapshot of surgical practice. No recurrences were observed during the 12-month follow-up period. Moreover, the authors reported that the use of fat grafts did not hinder oncologic imaging or postoperative surveillance, alleviating a common concern among clinicians.

While these findings are encouraging, they are tempered by the relatively short duration of follow-up. Oncologic recurrences-particularly in cases of low-grade salivary gland malignancies-may occur several years after initial treatment. Thus, long-term data are essential before definitive conclusions can be drawn.

Fiedler et al.'s survey offers valuable insight into current clinical practices and attitudes (1). It revealed a high level of acceptance of AFG among head and neck surgeons, with over 60% of respondents routinely using it for postcancer rehabilitation. Despite the widespread use, nearly half of the clinicians did not discuss oncologic risks with their patients-suggesting either confidence in the procedure's safety or a lack of robust data to guide informed consent practices. This discrepancy underscores the need for standardized guidelines and a deeper understanding of both real and perceived risks.

The narrative review by Drochioi et al. supports the clinical observations by highlighting AFG's biological potential and therapeutic applications (3). They detail how AFG improves vascularity, reduces fibrosis, and restores contour defects, especially in post-radiation fields. Their synthesis of fat graft biology emphasizes the multipotent and immunomodulatory properties of ASCs, yet the review ultimately concludes that current evidence does not support a causal link between AFG and oncologic recurrence.

The large series by Karmali et al. demonstrates that fat grafting can be performed safely even in a population exceeding 100 patients, with no recurrences arising at grafted sites. This reassures clinicians that AFG does not compromise oncologic surveillance or local control (4).

Griffin et al. further highlights the functional value of fat grafting, showing that lipotransfer effectively reduces radiation-induced fibrosis and restores volume, leading to marked improvements in esthetics and patient-reported quality of life. Importantly, their report of two recurrences occurring outside graft sites reinforces the view that AFG is unlikely to trigger local tumor reactivation (5).

Taken together, these findings emphasize that AFG not only appears safe from an oncologic standpoint but also contributes significantly to patient rehabilitation, particularly in improving post-radiation sequelae and psychosocial outcomes.

Biological Plausibility and In vitro Concerns

Despite the absence of clinical evidence of recurrence, the concern about oncologic risk remains biologically plausible. Several in vitro studies have demonstrated that ASCs can secrete factors such as vascular endothelial growth factor (VEGF), hepatocyte growth factor (HGF), and other cytokines that enhance angiogenesis and cellular proliferation. These properties, while beneficial for tissue regeneration, may also support neoplastic processes under certain conditions (9).

The stromal vascular fraction (SVF) of fat tissue, particularly when enriched or manipulated, may harbor a population of progenitor cells capable of interacting with dormant tumor cells. Studies in breast cancer models have shown mixed results-some indicating no oncologic risk, while others raise the possibility of enhanced tumor growth in co-culture or xenograft models (8 , 9). However, translating these findings to the head and neck domain remains speculative without robust, tumor-specific models.

Notably, none of the studies included in this review utilized cell-assisted lipotransfer (CAL), a technique involving fat graft enrichment with isolated ASCs. This distinction is important, as the oncologic behavior of standard AFG (minimally processed fat) may differ significantly from that of ASC-enriched grafts. As a precaution, ASC enrichment should be avoided in oncologic reconstruction until more data are available.

Limitations of the Current Evidence Base

This review identified several key limitations in the current literature:

The most significant limitation of this review is that only five studies met the inclusion criteria, underscoring the paucity of available evidence on this topic. This small evidence base limits the generalizability of our conclusions and highlights the urgent need for further clinical research.

- Sample Size and Study Design: Only one included study was clinical in nature, with a relatively small sample and short follow-up.

- Lack of Standardization: Variability in harvesting, processing, and grafting techniques complicates data interpretation (1).

- Absence of Long-Term Data: Most studies assess outcomes within 6 to 12 months, which is insufficient for robust oncologic safety assessment (2 , 3).

Limited Focus on Oncologic Outcomes: Many clinical reports focus on cosmetic or functional outcomes, often relegating oncologic considerations to secondary status.

These gaps hinder our ability to generalize findings across broader patient populations, tumor types, or reconstructive protocols.

Recommendations for Clinical Practice

Until more conclusive evidence becomes available, clinicians should adopt a cautious but pragmatic approach:

- Patient Selection: Fat grafting may be safest in patients with a low risk of recurrence and a sufficient disease-free interval.

- Informed Consent: The theoretical risks should be clearly communicated to patients, particularly in light of in vitro evidence.

- Surveillance: Imaging protocols should remain stringent, ensuring that grafted fat does not obscure local recurrence.

- Avoid Enrichment: Use of ASC-enriched fat should be restricted to research settings until oncologic safety is established.

Future Research Directions

Prospective, multicenter studies are urgently needed. These should include:

- Randomized controlled trials with long-term follow-up,

- Comparative studies of AFG versus alternative reconstruction techniques,

- Investigations into the molecular interaction between ASCs and residual cancer cells in the head and neck region,

- Standardization of graft preparation and placement protocols.

Such studies will enable evidence-based guidelines and bolster patient and clinician confidence in this promising technique.

## Conclusions

In conclusion, AFG appears to be a safe and effective technique for head and neck reconstruction in cancer patients, provided that appropriate precautions are taken. Further high-quality research is needed to develop standardized guidelines.

## Figures and Tables

**Table 1:** Table Summary of included studies on autologous fat grafting in head and neck cancer patients.

Author (Year)	Study Design	Population	AFG Technique	Follow up	Main Findings
Fiedler et al. (2021)	Survey of surgeons	45 surgeons (Germany, Austria, Switzerland, UK)	Various harvesting/processing methods	Not applicable	62% use AFG postcancer; no reported recurrences; variability in techniques
Boschetti et al. (2023)	Retrospective cohort	30 parotidectomy patients (8 malignant)	Dermis-fat graft from suprapubic region	12 months	No recurrences; satisfactory cosmetic outcomes; complications minor
Drochioi et al. (2019)	Narrative review	Oncologic craniofacial patients	Various techniques reported	Not specified	Highlighted regenerativepotential; no clinical evidence of recurrence
Karmali et al. (2018)	Retrospective series	116 patients; 190 procedures	Standard AFG	35.8 months	6 recurrences (none at graft sites); esthetic benefit
Griffin et al. (2019)	Retrospective cohort	38 patients	Lipotransfer (Coleman)	32 months	97% improved; 2 recurrences outside graft; QoL

AFG: Autologous fat grafting. QoL: Quality of life. Follow-up data not always reported. Findings are summarized as described in original publications.

## Data Availability

Declared none.
